# Editorial to the Special Issue “Eco-Physiological and Molecular Basis of Stress Tolerance in Plants”

**DOI:** 10.3390/biology12030485

**Published:** 2023-03-22

**Authors:** Lei Wang, Mohsin Tanveer

**Affiliations:** 1 State Key Laboratory of Desert and Oasis Ecology, Xinjiang Institute of Ecology and Geography, Chinese Academy of Sciences, Urumqi 830011, China; 2 Tasmanian Institute of Agriculture, University of Tasmania, Hobart, TAS 7000, Australia

Farmers are currently facing the challenge of producing sufficient crop yield. Two factors contributing towards such difficulty are the availability of agricultural land to use for crop cultivation, which is currently around 1.5 billion ha (11%) of the total land surface area, and secondly, climate change. The latter is more detrimental, as the onset of different environmental setbacks is shifting the available agricultural land into barren or saline land. Thus, there is a dire need to understand the impacts of climate change on the crop production along with improving the performance of crops under different biotic or abiotic stress conditions. In addition, the ongoing increase in the world’s population, which is expected to grow to 9.6 billion people by 2050 [1], is further exaggerating and putting pressure on the need to increase crop production and meet food demand. 

Ongoing climate change will introduce inevitable episodes of unfavourable weather conditions, which will affect crop production worldwide. Plants are exposed to different abiotic stresses such as extreme- or low-temperature regimes, or periods of water shortage, soil salinity, and heavy metal pollution [2,3,4]. All these abiotic stresses dramatically reduce crop production worldwide. Nonetheless, such abiotic stresses also lead to the introduction of biotic stress: for example, excessive rainfall or heavy dew leads to the infestation of Fusarium head blight on small-grain cereals [5]. At the global level, several research groups are working to improve stress tolerance in plants in order to cope with current climate change and food demand; however, a lot of improvement is still required to enhance stress tolerance. Recently, it has also been argued that focusing on one trait or expressing one particular gene does not offer satisfactory results under different abiotic stress; thus, there is a need to combine agronomic and genetic techniques to enhance stress tolerance overall in plants. 

This Special Issue presents recent research relating to the improvement of stress tolerance in plants. Seed germination is the first and most susceptible growth stage in response to stress conditions, and any alteration in growth conditions significantly reduces seed germination [6]. To enhance our understanding of improving seed germination under drought stress, Zhao and his colleagues examined the genetic diversity and performed quantitative genetics analyses to identify loci associated with the drought tolerance of seed germination in 410 accessions of a germplasm diversity panel for soybean [7]. They uncovered significant differences among the diverse genotypes for four growth indices (i.e., relative germination energy, relative germination rate, germination stress index, germination drought tolerance index, and membership function value) and five drought-tolerance indices, which revealed abundant variation among genotypes upon drought stress, and for genotype × treatment effects. They used 158,327 SNPs and then performed GWAS, identifying 41 candidate genes for drought tolerance indices during seed germination and 26 SNPs linked with drought tolerance indices at the germination stage [7]. The obtained results provide a molecular understanding of the regulation of seed germination under drought stress. 

Photosynthesis is the second most susceptible physiological mechanism in plants in response to stress conditions. Abiotic stress conditions reduce photosynthesis by altering leaf gas exchange or by disrupting chlorophyll biosynthesis [8] and targeting these physiological mechanisms may improve overall crop productivity under abiotic stress conditions. Among various abiotic stresses, high-temperature (HT) stress reduces photosynthesis primarily by reducing the chlorophyll contents associated with HT-stress-induced alteration in chloroplast structure, leaf gas exchange, and the enhancement of pheophytinase activity, and ROS production [9]. However, the degree of HT-stress-induced reduction varied among different cultivars; thus, it is important to understand the physiological and molecular regulation of photosynthesis under HT stress. In a study, Li and his colleagues examined the physiological basis of HT-stress-induced reduction in photosynthesis in kiwifruit [10]. They found that a temperature exceeding 44.5 °C was detrimental to kiwifruits; nonetheless, kiwifruit cultivars with different ploidy levels (diploid and hexaploid) were found to be sensitive to HT stress, while tetraploids showed relatively higher HT tolerance, suggesting a positive correlation between polyploidy and variation in HT-induced reduction in photosynthesis [10].

In another study, the role of Basic Helix–Loop–Helix (bHLH) transcription factor was examined, and *OsbHLHq11* was found to regulate photosynthesis efficiency by regulating chlorophyll contents in rice [11]. The authors measured leaf colour, chlorophyll content, and chlorophyll fluorescence; they also performed QTL mapping and found a major QTL was related to chlorophyll contents on chromosome 11. They observed that *OsbHLHq11* was highly expressed in cultivars with low chlorophyll contents and suggested that *OsbHLHq11* can be used as a new genetic resource to breed rice cultivars with better photosynthesis efficiency [11]. 

As plants are sessile organisms, they must regulate their growth and adapt different responses in order to survive under different hostile conditions. Such adaptive responses include the activation of a defence system or stress regulatory mechanisms which are regulated by different plant growth regulators or hormones. Hormones such as jasmonates, brassinolides, auxin, and others regulate different adaptive responses in plants, either at the site of their synthesis or following their transport elsewhere in the plant. In this regard, three papers are included in this special issue. The first one was published by Ye and his colleagues, who characterised the functionality of the JASMONATE ZIM-DOMAIN (JAZ) gene family in pineapple [12]. JAZ proteins are negative regulators of jasmonate signalling in response to abiotic and biotic stress in plants. They identified 14 JAZ genes in pineapple, which were highly upregulated and expressed during plant development in response to different abiotic stress conditions including salinity, high and low temperature, osmotic stress, and phytohormone application. Moreover, a bimolecular fluorescence complementation analysis showed that these JAZ proteins could interact with other stress regulatory proteins, such as MYC2, NINJA, and JAM1 to regulate abiotic stress tolerance in plants [12]. Thus, targeting JAZ proteins could regulate abiotic stress tolerance in plants. Likewise, Yu and his colleagues examined the role of ethylene-responsive element (AP2/ERF) in almond under freezing stress [13]. They performed a genome-wide analysis of AP2/ERF and then performed an expression analysis of AP2/ERF during freezing stress. The obtained results identified a total of 136 PdAP2/ERF genes in the almond genome and found 96 PdAP2/ERF genes expressed among four tissues, leaves, flowers, flower buds, and fruitlets of almond. Moreover, the cis-acting elements analysis showed that PdAP2/ERF members are widely involved in various processes, such as growth and development, hormone regulation, and stress response. Thus, this study provides in-depth detail of the role of ethylene responses elements in regulating almond dormancy under freezing stress. Cardoso and his colleagues [14] emphasized that gene diversification among domesticated cultivars and their wild parents may be an important aspect in understanding the role of hormones in regulating abiotic stress tolerance in plants [14]. For instance, they examined the gene expression of PIN gene family in the domesticated olive tree and its wild relative [14]. PIN proteins are responsible for auxin transport and play a crucial role in activating adaptive responses to stress conditions; however, the expression patterns could be varied among different plant species and cultivars. Cardoso et al. [14] identified a differential pattern of gene expression among domesticated olive tree and its wild relatives under wounding, biotic stress (infection with *V. dahliae*), and abiotic stress (cold exposure), thus highlighting the role of PIN genes in conferring stress tolerance in olive trees. 

The carbohydrates metabolism involves the breakdown of carbohydrates into glucose to supply energy to living cells. This process is highly sensitive to abiotic stress conditions; thus, it is important to understand the physiological regulation of this process under abiotic stress conditions. Moreover, invertase enzymes are amongst the most important enzymes that regulate the carbohydrate metabolism (i.e., the conversion of sucrose into fructose and glucose) and sugar utilization. Considering this, a study by Abbas and his colleagues [15] is included in this special issue. They performed genome wide identification and an expression analysis of the invertase gene family in sweet potato under biotic (*Phytophthora infestans*) and abiotic (salinity, mannitol, hormonal, and HT) stress conditions. They found a total of 11 invertase genes belonging to acidic invertase enzymes and eight genes belonging to alkaline invertase enzyme, indicating their functional divergence [15]. They also found that the regulation of invertase proteins was highly tissue-specific, and their expression was differently regulated by different stress conditions. Thus, this study provides comprehensive data about the role of the invertase gene family in the regulation of sugar metabolism and abiotic stress tolerance in potato. 

Given that the above studies focus on the genetic and physiological regulation of abiotic stress tolerance in plants, we have also included three studies examining the exogenous application of plant growth regulators and Rhizobacteria in conferring abiotic stress tolerance in plants. For instance, Khan and his colleagues [16] examined the physiological role of the exogenous application of 24-Epibrassinolide (EBL) in tobacco under drought stress. They found that drought stress significantly arrested the tobacco growth by changing the leaf anatomy and increasing ROS production; however, the exogenous application of EBL conferred drought tolerance in tobacco by increasing leaf thickness to reduce transpiration and activation of the antioxidant defence system [16]. They also showed that EBL application not only upregulated the expression of genes involved in brassinolide signalling, but also upregulated the genes involved in the indole acetic acid signalling pathway, suggesting a highly orchestrated network of hormonal signalling pathways to confer drought tolerance in tobacco. Likewise, Ahmad and his colleagues showed that the combined application of melatonin and KNO3 improved tolerance to waterlogging stress in maize [17]. They observed that waterlogging stress reduced root biomass, plant height, and leaf area per plant, and induced oxidative stress by increasing ROS production. Nonetheless, melatonin application along with KNO3 conferred waterlogging stress in maize by increasing the activation of antioxidant defence system, osmolytes accumulation, and improving nitrogen metabolism, thus improving maize growth and development [17]. We realized that such a response could be highly sensitive to the temporal and maize growth stage; thus, future studies should consider this point while further studying the stress regulatory role of melatonin in plants. In addition to these, Heo and his colleagues [18] showed that the application of Rhizobacteria Burkholderia contaminants AY001 was very promising in controlling the infestation of *Fusarium Wilt* and *Bacterial Speck* disease in tomato [18]. They found that the application of AY001 resulted in (i) a higher expression of several plant growth-promoting rhizobacteria (PGPR)-related traits such as zinc and phosphate solubilization, nitrogen fixation, IAA biosynthesis; (ii) a higher production of secondary metabolites; and (iii) the induction of systemic acquired resistance [18]. They concluded that the application of AY001 could be a safe and very promising strategy to control pathogenic infestation and can be used as a sustainable biocontrol agent. Finally, the last research article included in this Special Issue showed that the application of waste-derived organic amendments resulted in the accumulation of heavy metals in garlic, thus imposing serious health problems [19]. Generally, it is considered that waste-derived organic amendments are a cheap and sustainable way to ameliorate soil-related problems such as soil fertility or soil salinity; however, the authors showed that such amendments could increase the accumulation of other heavy metals in soil, which can be taken up by plants and reach our table.

Technological advancement also plays a significant role in deciphering and understanding the complex stress tolerance mechanisms in plants. Different techniques have different advantages and disadvantages; however, their adoption solely depends on the aim and objective of the investigation. Moreover, the development of breeding tools or long-term breeding programs require budgets and is time consuming; thus, an article by Zhang et al. [20] is also included in this special issue. They examined and used the heavy ion beam mutagenesis technique to develop and screen saline–alkaline-tolerant rice mutants. The obtained results showed that heavy ion beam radiation is an effective method for breeding new saline–alkaline-tolerant rice cultivars, and the selected mutant lines show excellent production performance under saline–alkaline stress [20]. Thus, this study provided new insights to develop rice cultivars for saline and alkaline soils.

Three review articles are also included in this Special Issue. Boorboori and Zhang [21] reviewed the physiological role of *Serendipita indica* (*Piriformospora indica*) in improving plant resistance to drought and salinity stress [21]. They highlighted the impact of this fungus in tackling environmental stresses and enhancing agricultural productivity, as *Serendipita indica* is phylogenetically like arbuscular mycorrhizal fungi and can colonize under different stress conditions and coexist with more than 200 plant species. Moreover, this fungus can be easily grown in artificial medium and can be used as a promising biofertilizer [21]. According to this review, *Serendipita indica*-mediated stress tolerance in plants results from several complementary physiological mechanisms, including (i) higher root proliferation and root biomass, (ii) higher nutrient uptake, (iii) the activation of genes involved in microtubule-based processes, (iv) decreasing stomatal conductance under drought stress, (v) and the activation of the antioxidant defence system (for a detailed review, see Boorboori and Zhang [21]). In the second review article, Shanker and his colleagues discuss the role of elevated CO_2_ in ameliorating the drought-induced negative effects on plant growth and yield, as well as the possible ways by which there can be effective adaption to crops in the changing climate scenario [22]. They discussed the effects of elevated CO_2_ and drought conditions on stomatal dynamics, the ABA conundrum, and dry matter production in plants, and these effects were varied among C3 and C4 plants. Overall, this review article provides an in-depth explanation of how CO_2_ and drought stress collectively regulate plant growth and development and what strategies we can consider while developing new varieties for the future. The third and final review article published in this special issue provides an insight into abiotic stress and influx tolerance mechanisms in plants under salinity stress [23]. The authors discussed and highlighted the role of osmolytes, signal transduction, and hormonal signalling in conferring salinity tolerance in plants. They also briefly reviewed the recent advancement in understanding the role of the SOS pathways as salt sensing mechanisms in plants under salinity stress.

In summary, this special issue collates a selection of papers emphasizing the importance of different physiological and molecular aspects of stress tolerance in plants. The combination of agronomic and molecular research can improve overall crop production. We hope that this Special Issue will improve our knowledge of the intricacy of stress physiology and crop production.

## Data Availability

Not applicable.
